# New Functional Food for the Treatment of Gastric Ulcer Based on Bioadhesive Microparticles Containing Sage Extract: Anti-Ulcerogenic, Anti-*Helicobacter pylori*, and H^+^/K^+^-ATPase-Inhibiting Activity Enhancement

**DOI:** 10.3390/foods14152757

**Published:** 2025-08-07

**Authors:** Yacine Nait Bachir, Ryma Nait Bachir, Meriem Medjkane, Nouara Boudjema, Roberta Foligni

**Affiliations:** 1 University of Blida 1, BP 270 Route Soumâa, Blida 09000, Algeria; 2 Elbe Klinikum Stade, Bremervörder Straße 111, 21682 Stade, Germany; naitbachir.r@gmail.com; 3 University of Algiers 1, BP 2 Rue Didouche Mourad, Algiers 16000, Algeria; 4 Department of Biology, Institute Life and Natural Sciences, University Center Nour Bachir El Bayadh, El Bayadh 32000, Algeria; medjkanemeriem@yahoo.fr; 5 Laboratory of Natural Bio-Resources, Department of Nutrition and Food Sciences, Faculty of Life and Natural Sciences, Hassiba Benbouali University, Chlef 02010, Algeria; 6 University of Science and Technology Houari Boumediene, Bab Ezzouar, Algiers 16000, Algeria; boudjemanouara@gmail.com; 7 Department of Human Sciences and Promoting of the Quality of Life, San Raffaele University Rome, Via Val Cannuta 247, 00166 Rome, Italy

**Keywords:** microencapsulation, emulsion–coacervation, anti-ulcerogenic, anti-*Helicobacter pylori*, H^+^/K^+^ ATPase inhibition, in vitro dissolution, bioadhesion

## Abstract

*Salvia officinalis* is an aromatic plant of Mediterranean origin traditionally used to treat inflammatory, cardiovascular, endocrine, and digestive diseases. In this work, the ability of the *Salvia officinalis* extract in the treatment of gastric ulcers was evaluated, and an innovative administration system was proposed to increase the therapeutic effect of this plant. *Salvia officinalis* ethanolic extract was prepared and analyzed by HPLC/UV-DAD and encapsulated in a matrix based on gelatin and pectin using an emulsion–coacervation process. The prepared microcapsules were analyzed by laser particle size, optical microscopy, in vitro dissolution kinetics, and ex vivo bioadhesion. In order to determine the action mechanism of *Salvia officinalis* extract, in the treatment of gastric ulcer, the in vivo anti-ulcerogenic activity in rats, using the ulcer model induced by ethanol; the in vivo anti-*Helicobacter pylori* activity; and in vitro inhibitory activity of H^+^/K^+^-ATPase were carried out. These three biological activities were evaluated for ethanolic extract and microcapsules to determine the effect of formulation on biological activities. Ethanolic extract of *Salvia officinalis* was mainly composed of polyphenols (chlorogenic acid 7.43%, rutin 21.74%, rosmarinic acid 5.88%, and quercitrin 14.39%). Microencapsulation of this extract allowed us to obtain microcapsules of 104.2 ± 7.5 µm in diameter, an encapsulation rate of 96.57 ± 3.05%, and adequate bioadhesion. The kinetics of in vitro dissolution of the extract increase significantly after its microencapsulation. Percentages of ulcer inhibition for 100 mg/kg of extract increase from 71.71 ± 2.43% to 89.67 ± 2.54% after microencapsulation. In vitro H^+^/K^+^-ATPase-inhibiting activity resulted in an IC50 of 86.08 ± 8.69 µM/h/mg protein for free extract and 57.43 ± 5.78 µM/h/mg protein for encapsulated extract. Anti-*Helicobacter pylori* activity showed a similar Minimum Inhibitory Concentration (MIC) of 50 µg/mL for the extract and microcapsules. *Salvia officinalis* ethanolic extract has a significant efficacy for the treatment of gastric ulcer; its mechanism of action is based on its gastroprotective effect, anti-*Helicobacter pylori*, and H^+^/K^+^-ATPase inhibitor. Moreover, the microencapsulation of this extract increases its gastroprotective and H^+^/K^+^-ATPase-inhibiting activities significantly.

## 1. Introduction

Gastric ulcer is a disease that represents an annual treatment cost of several billion dollars, and this disease is mainly due to *Helicobacter pylori* (HBP). Moreover, the secondary complications include cancer caused by toxins produced by pathogenic bacteria [1]. Treatment of ulcer based on the elimination of bacteria with antibiotic therapy promotes healing using proton pump inhibitors [1,2].

Bioactive molecules of plant origin have been used through the years for the treatment of human or animal pathologies. Most medicinal plants are traditionally used for preparing infusions or drinks; however, with the development of the pharmaceutical industry and the expansion of the natural medicine market, high-performance pharmaceutical forms were proposed for the vectorization of these natural bioactive molecules [3,4,5,6].

*Salvia officinalis* is an aromatic plant of Mediterranean origin. It is traditionally used for the treatment of several diseases, and it has anti-microbial, anti-cancer, anti-HIV, cytotoxic, antipyretic, anti-ulcerogenic, antidiabetic, and hypolipemic activities [7,8,9,10]. These therapeutic properties of sage are due to the presence of volatile (essential oil) and non-volatile bioactive molecules (polyphenols) [7,8,9,10,11]. In the literature, there are only three studies that showed the gastroprotective activity of *Salvia officinalis* [12,13,14]. Other studies have shown that *Salvia officinalis* has anti-HBP activity [15,16,17,18], which makes this plant a potential candidate for gastric ulcer treatment.

Microencapsulation is a set of technologies that allows the preparation of particles between 1 μm and 1 mm in size, made of an encasing material containing an active substance. Encapsulated materials come from a wide variety of sources (pharmaceuticals, cosmetic ingredients, food additives, microorganisms, cells, or chemical reaction catalysts), and coating materials are often polymers of natural or synthetic origin. There are two types of microparticle morphologies: microcapsules that are a reservoir of particles consisting of an active material core surrounded by an encasing material membrane, and microspheres that are particles consisting of a continuous network of encapsulating material forming a matrix in which the finely dispersed active material is found [19,20]. The interest in microencapsulation, in general, is to ensure the protection and stabilization of an active substance [21], to improve the presentation of a substance (hide a taste, odor, or color), to carry out an adapted form such as the transformation of a liquid active substance into a powder, or to modify and control the release profile of an active substance to obtain a controlled release [22]. In the field of therapeutic design, microencapsulation has often been used to modify the pharmacokinetics of drugs [23] in order to increase their in vitro dissolution kinetics and in vivo bioavailability, which corresponds to the increase in therapeutic effect of the drug.

The originality of the present research lies in increasing the anti-ulcerogenic activity of *Salvia officinalis* extract microencapsulation. The work has been carried out in three steps:

The first step was the extraction of bioactive molecules from *Salvia officinalis* leaves and the determination of their chemical composition by HPLC/UV-DAD. The second step was the extract’s microencapsulation in a gelatin/pectin-based system using a complex double emulsion–coacervation process. The microcapsules obtained were characterized by laser particle size and optical microscopy, in vitro kinetics dissolution, and ex vivo bioadhesion. The third step was the treatment mechanism study of gastric ulcer by *Salvia officinalis* extract before and after microencapsulation by evaluating in vivo anti-ulcerogenic, in vitro anti-HBP, and in vitro H^+^/K^+^-ATPase-inhibiting activities.

## 2. Materials and Methods

### 2.1. Materials

#### 2.1.1. Plant Material

*Salvia officinalis* leaves were collected at the flowering stage (Saturday, 11 February 2015) in Roudah, Ouled Sidi Brahim, Bouira city, at 170 km from Algiers, Algeria. (Geonames-ID: 2485311, Latitude: 36.36748, Longitude: 3.70329, Altitude: 801). The plant was identified at the herbarium of the NIA (National Institute of Agronomy, Algiers, Algeria). Before its use, the plant underwent washing, drying, crushing, and sieving at 500 µm.

#### 2.1.2. Chemical Reagents

Gelatin type B (of bovine origin), pectin, and Folin–Ciocalteu reagent were purchased from Sigma Aldrich, St. Louis, MO, USA. Virgin olive oil from organic farming was offered by the growers of ROUDAH. Lecithin was provided by the Belat food processing company. Formaldehyde, hydrochloric acid, ethanol, phosphoric acid, acetonitrile, sodium hydroxide, sodium carbonate, and distilled water were of analytical grade.

### 2.2. Preparation and Characterization of the Extract

#### 2.2.1. Soxhlet Extraction of Bioactive Molecules

A total of 20 g of plant powder was inserted into a cellulose extraction cartridge (Albert labscience^®^, Dassel, Germany) and placed in a Soxhlet with 300 mL of ethanol. After 120 min of extraction, the organic solvent was evaporated under vacuum using a Heidolph^®^ rotavapor (Schwabach, Germany), and the extract was recovered. The extraction yield was calculated by the following equation:

$$R\left(\%\right)=\frac{m_{\mathrm{ext}}}{m_{v}}\times 100$$

where ‘R’ is the yield (%), ‘m_ext_’ is the mass of the extract after solvent evaporation (g), and ‘m_v_’ is the initial plant mass in the cartridge (g).

#### 2.2.2. HPLC-UV/DAD Analysis of the Extract

The analysis was performed using a WATERS-type HPLC (Milford, MA, USA) device with a UV/DAD detector. The method used was that described by Gîrd et al. and Nait Bachir et al. [11,24]. Briefly, the stationary phase used was Nucleosil—C18 (Sigma Aldrich); 25 × 0.4 mm i.d.; and 5 μm particle. The mobile phase used was a mixture of two solvents (water and phosphoric acid at 999:1 (*v*/*v*)—solvent A; acetonitrile: solvent B). The gradient used was 90%A/10%B, 0 min; 90 to 78% A/10 to 22%/B, 0–13 min; 78 to 60%A/22 to 40%/B, 13–14 min; 60%A/40%B, 14–20 min. The flow rate was 1.5 mL/min, the injection volume was 20 μL, and the follow-up wavelength was 310 nm.

The identification of separate compounds was carried out by comparing retention times of peaks obtained with the retention times of standards previously analyzed under the same operating conditions, and standards used were chlorogenic acid (5.42 min), rutin (15.63 min), rosmarinic acid (16.70 min), and quercitrin (18.31 min).

### 2.3. Preparation and Characterization of Microcapsules

#### 2.3.1. Preparation of Microcapsules

Microcapsules were prepared using the emulsion–coacervation technique. The process was carried out in two main steps: first step—emulsion preparation; second step—emulsion–coacervation using gelatin and pectin for the formation of the microcapsule membrane.

Step 1: A water-in-oil (W/O) emulsion was formed using the method described in [25] with several modifications. Briefly, a solution of 0.5 g of *Salvia officinalis* extract in 5 mL of distilled water was mixed with 10 mL of olive oil containing 0.75 g of lecithin (corresponding to 3% of total solids used in the formulation). After 15 min of stirring, the primary emulsion W/O was obtained.

Step 2: Complex emulsion–coacervation was achieved by combining several methods [26,27,28] with modifications. Briefly, a double W/O/W emulsion was formed by mixing the previously prepared W/O emulsion with 28.5 mL of 5% gelatin solution at a temperature of 40 °C (protein denaturing temperature). After 15 min of stirring, 11.5 mL of the 5% pectin solution at a temperature of 40 °C was added, pH of the obtained system was adjusted to 3 (isoelectric pH of protein). The system was cooled to 5 °C (freezing of microparticles by cold), and then 1 mL of formaldehyde was added and stirred for 30 min. Finally, pH of the system was adjusted to 9 using a solution of sodium hydroxide at 20%, with agitation for 2 h. Prior to filtration and freeze-drying, the microcapsules were washed by rinsing them three times with ethanol to remove surface-adsorbed polyphenols, followed by three rinses with distilled water to eliminate unbound water-soluble polymers present on the microcapsule surface.

#### 2.3.2. Encapsulation Rate

Encapsulation rates were determined using the following equation [13]:

$$Encapsulation\;rate=\;\frac{\;Encapsulated\;polyphenol\;amount\;\left(g\right)}{0.5}\cdot 100$$

The amount of polyphenols initially introduced was known (representing 0.5 g), and the amount of encapsulated polyphenols was determined by the determination of the total polyphenol amount contained in freeze-dried microcapsules.

A total of 100 mg of microcapsules was finely ground and mixed with 100 mL of ethanol; after 1 h of stirring, the solution was filtered using a syringe microfilter with a pore diameter of 0.2 µm. The total amount of polyphenols was determined by the Folin–Ciocalteu method [29,30,31].

The assay was performed using a SHIMADZU^®^-type UV–Visible spectrophotometer (Kyoto, Japan), with 0.5 mL of each sample solution mixed with 2.5 mL of 20% *w*/*v* sodium carbonate solution and 2.5 mL of Folin–Ciocalteu reagent at 10% *v*/*v*. After 2 h of incubation at room temperature, the absorbance of the solutions was determined at a wavelength of 768 nm. The quantification was performed according to a calibration curve established under the same conditions with extract solutions of different concentrations (from 0 to 50 μg/mL). All the measurements were carried out in triplicate at 25°.

#### 2.3.3. Particle Size Analysis

The average size of the prepared suspension particles was determined by a FRITSCH ANALYSETTE 22 MicroTec plus laser granulometer (Idar-Oberstein, Germany). The average diameter was calculated using the following equation:

$$D_{M}=\frac{\sum_{i=1}^{n}f_{i}\cdot x_{i}}{\;\sum_{i=1}^{n}f_{i}}$$

where ‘D_M_’ is the arithmetic mean diameter, ‘n’ is the number of classes fractionating the sample, ‘x_i_’ is the representative diameter, and ‘f_i_’ is the frequency. All the measurements were tripled and performed at 25 °C.

#### 2.3.4. Microscopic Observation

Microscopic observation of the resulting microcapsules was performed using an Olympus^®^ BX60-type optical microscope (Tokyo, Japan).

#### 2.3.5. In Vitro Dissolution Kinetics Study

The dissolution kinetics was performed using a dissolution apparatus, ‘Electrolab TDT-08L dissolution apparatus’ (Mumbai, India), using the paddle system. The test of dissolution was performed with 250 mL of medium at 37 ± 0.5 °C with a stirring force at 100 rpm.

Studies of the dissolution kinetics of the *Salvia officinalis* extract and preparation of microcapsules were carried out in three different media: gastric medium (solution of HCl at 0.1 M, pH = 1.2), intestinal medium (phosphate buffer, pH = 6.8), and blood medium (phosphate buffer, pH = 7.4).

The microcapsules (equivalent to 20 mg of extract) were placed in a dissolution vessel. A sample of 1 mL was made at different times (15 min, 30 min, 1 h, 2 h, 3 h, 4 h, 5 h, 6 h, 12 h, and 24 h), and each time the sample was replaced by 2 mL of medium.

The sample was filtered using a 0.1 µm millipore syringe filter, and then its total polyphenol concentration was determined by the Folin–Ciocalteu method previously described in Section 2.3.2 above. The dissolution was calculated using the following equation:

$$D_{t}=\frac{C_{t}V}{M}$$

where ‘D_t_’ is the dissolution at the time ‘t’, ‘C_t_’ is the concentration of polyphenols in 1 mL of solution taken at each time interval, ‘V’ is the volume of medium in the dissolution vessel (250 mL), and ‘M’ is the total amount of polyphenols introduced into the dissolution apparatus. All the experiments were performed in triplicate.

#### 2.3.6. Measurement of Ex Vivo Bioadhesion:

Bioadhesion of microparticles was determined by the ex vivo method described by [28] (Saravanan and Rao, 2010) with modifications. A total of 10 cm of spawning rat gut was sectioned and washed with isotonic saline water (9/1000 NaCl). The nanoparticles were placed on the surface of the intestinal mucosa, and then the system was incubated at 37 °C. After incubation, the parts of the intestine were rinsed with phosphate buffer at a flow rate of 20 mL/min for 4 h using a peristaltic pump. Photographs of the microcapsules placed on the intestine were taken after 1 h, 2 h, 3 h, and 4 h of rinsing, and the surface of the intestine lined by the microcapsules was determined by image processing using the software image-J NIH version (Bethesda, MD, USA). The percentage of microcapsules remaining after rinsing was calculated by the following equation:

$$PR=\frac{S_{t}}{S_{0}}\cdot 100$$

where ‘PR’ is the percentage of the remaining microcapsules, ‘S_t_’ is the surface of the intestine lined by microcapsules at time ‘t’, and ‘S_0_’ is the surface of the intestine lined by microcapsules before rinsing begins. All the experiments were performed in triplicate.

### 2.4. Evaluation of Biological Activities

#### 2.4.1. In Vivo Evaluation of Anti-Ulcerogenic Activity

##### Animal Material and Experimental Design

The objective of this study is to evaluate the effect of microencapsulation on the anti-ulcer activity of the extract.

Rats were obtained from the Pasteur Institute of Algeria (IPA, Algiers, Algeria). They were healthy and had not undergone any prior experimental procedures before the start of this study.

The study was conducted at the Animal Experimentation Station of the University of Saad Dahleb Blida 1 (Blida, Algeria), and all animal experiments were carried out in strict accordance with the European Union Directive code: 2010/63/EU on the protection of animals used for scientific purposes.

A total of 30 Wistar rats (Rattus norvegicus) were used in this study. They were divided into 5 groups of 6 rats each. The animals were 8 to 10 weeks old, with body weights ranging from 180 to 200 g.

The rats were observed daily during a two-week acclimatization period to ensure their general health status and proper adaptation to the experimental conditions. They were housed under standardized conditions (temperature 22 ± 2 °C, relative humidity 55 ± 10%, and 12 h light/dark cycle) with free access to food and water (ad libitum).

In this study, no exclusion criteria were defined a priori, and no animals or data points were excluded from the analysis, as the protocol was applied with 100% reproducibility.

The animals were kept fasted for 18 h before experimentation. Protocol used for the evaluation of anti-ulcerogenic activity as reported in [32] with some modifications. At t0 each group was treated by intragastric gavage as shown in Table 1. One hour after administration of the treatment, the rats were gavaged with 1 mL of ethanol.

One hour after administration of ethanol, the rats were sacrificed by cervical dislocation under ketamine anesthesia at 50 mg/mL, and the stomachs were immediately recovered, opened by the large curvature, and rinsed with isotonic physiological water (aqueous solution of 9/1000 of NaCl).

Photographs of the stomachs were taken to determine ulcers and inhibition percentages, and then they were directly introduced into formalin for the histopathological study.

##### Determination of Ulcer and Inhibition Percentages

The photographs taken from the different stomachs were analyzed by image processing using the image-J software to determine the percentages of ulcer (PU) using the following equation:

$$PU=\frac{ulcer\;surface}{total\;surface\;of\;the\;stomach}\cdot 100$$

The percentages of ulcer inhibitions were calculated for each group using the following equation:

$$PI=\frac{PU_{\mathrm{negative}\;\mathrm{control}\;}-PU_{treated\;group}}{PU_{\mathrm{negative}\;\mathrm{control}\;}}\cdot 100$$

##### Histopathological Study

The stomachs were recuperated, fixed in formalin for 48 h, dehydrated, and then histological sections of 6 µm were obtained. Finally, the blades were colored by an HE coloration. The prepared slides were observed under an optical microscope to evaluate different histological characteristics of the ulcer according to the method described by [33] with some modifications. The five histological characteristics were as follows:

1. Epithelial desquamations.
2. Destruction of glands.
3. Swelling of the sub-mucous membrane.
4. Infiltration of eosinophils.
5. Bleeding.

The scores for histological characteristics of each batch of stomachs were assigned using the following system: absence (-), slight presence (+), presence (++), and strong presence (+++).

#### 2.4.2. H^+^/K^+^-ATPase-Inhibiting Activity

##### Isolation of H^+^, K^+^ ATPase Enzyme

The H^+^, K^+^ ATPase enzyme was isolated from the gastric mucous membranes of rabbits using the ultracentrifugation and gradient separation methods described in [34]. A part of the stomach body was severed, and the mucous membrane that covers it was gently scraped. A total of 10 g of this mucous membrane was mixed with 100 mL of pH 7.4 buffer composed of 250 mM of sucrose, 2 mM of MgCl_2_, 1 mM of ethylene glycol tetracetic acid, and 2 mM of Hepes.

The mixture was homogenized using a pestle and then centrifuged at 20,000 RPM for 20 min. The supernatant was recovered and centrifuged at 100,000 RPM for 60 min. The base was recovered and resuspended in a buffer solution, pH 7.4, with 30% sucrose, and then centrifuged at 100,000 RPM for 60 min. The sedimented membranes were used as the vesicular fraction containing gastric H^+^/K^+^ ATPase. All the manipulations were performed at temperatures less than or equal to 4 °C (between 2 and 4 °C). The resulting product was freeze-dried, frozen, and stored at −80 °C until use.

The protein assay in the product was determined using the method described in [35] using bovine serum albumin as a standard.

##### Determination of H^+^, K^+^ ATPase-Inhibiting Activity

Inhibiting activity of H^+^, K^+^ ATPase was evaluated in vitro using the method described in [36]. Briefly, 1 mL of a reaction medium containing 2 mM/mL MgCl_2_, 40 mM/mL Tris-HCl, 1 mM/mL ATP, and 20 mM/mL KCl was mixed with 5 µg H^+^/K^+^ ATPase gastric (in the presence or absence of free or encapsulated *Salvia officinalis* extract). The media was incubated at 37 °C for 20 min, and the reaction was inhibited by adding 1 mL of a cold solution of 10% trichloracetic acid. The colorimetric determination of inorganic phosphate released by the enzyme was performed using the protocol described by [37].

The IC50 was determined after H^+^, K^+^ ATPase-inhibiting activity evaluation in a range of *Salvia officinalis* extract solutions diluted in dimethylsulfoxide (25, 50, 75, 100, 125, and 150 µg/mL). The IC50 of omeprazole (used as a standard) was determined in the same way. The concentration of dimethylsulfoxide should not exceed 1% of the reaction medium (not to affect enzymatic activity). All the experiments were carried out in triplicate.

#### 2.4.3. Anti-*Helicobacter pylori* Activity

The anti-*Helicobacter pylori* activity was evaluated in vitro using the agar dilution method, described by the National Committee for Clinical Laboratory Standards [38]. The *Helicobacter pylori* strain used was derived from an endoscopy clinical isolation found in the microbiology laboratory of the Mustafa Bacha Hospitalo-University Centre (Algiers, Algeria). An H. pylori suspension was prepared in BHIB at an opacity of 3 to 4 according to McFarland standards (approximately 109 CFU/mL).

For the determination of the MIC, a series of dilutions of free or encapsulated *Salvia officinalis* extract in Mueller–Hinton agar with 10% horse blood were performed (100, 50, 25, 12.5, 6.25, 3.125, 1.56, 0.78, 0.39, 0.2, 0.1, 0.05, 0.02, and 0.01 µg/mL), and the prepared agar was poured directly into Petri dishes.

After cooling and solidification, the Petri dishes were inoculated with the H. pylori suspension and then incubated in a microaerophilic environment (5% O_2_, 10% CO_2_, and 85% N_2_) in an anaerobic container using BD GasPak EZ Pouch Systems for 48 h.

The lowest concentration of the *Salvia officinalis* extract showed no growth, which was identified as MIC. All the experiments were performed in triplicate.

### 2.5. Statistical Analysis

Data were expressed as mean ± standard deviation (SD). Statistical analysis was performed using Microsoft Excel with the ExcelStat add-in (Addinsoft, Paris, France). Comparisons between the results were made using one-way ANOVA, followed by the Bonferroni post hoc test. A *p*-value of <0.05 was considered statistically significant.

## 3. Results and Discussion

### 3.1. Preparation and Characterization of the Extract

**Figure 1:**
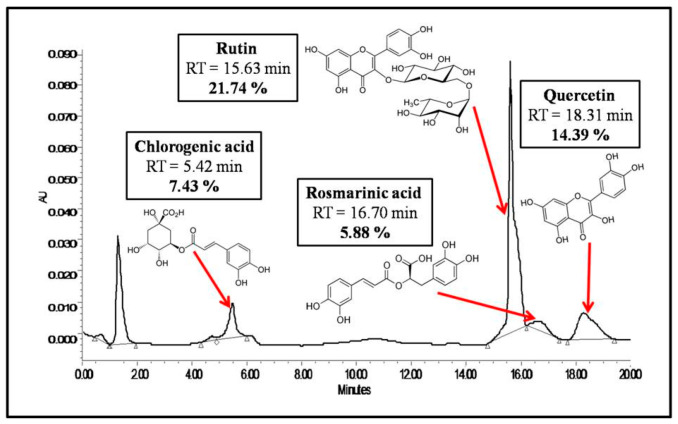
HPLC chromatogram of *Salvia officinalis* ethanolic extract.

The efficiency of bioactive *Salvia officinalis* molecules extraction was 24.16 ± 2.05%. The HPLC/UV-DAD analysis identified four phenolic compounds (two phenolic acids and two flavonoids). The extract was composed of chlorogenic acid (7.43%), rutin (21.74%), rosmarinic acid (5.88%), and quercitrin (14.39%); the identified compounds correspond to a total of 49.44% of the extract. The HPLC chromatogram is shown in Figure 1.

### 3.2. Preparation and Characterization of Microcapsules

#### 3.2.1. Encapsulation Rate and Structure of Obtained Microcapsules

The encapsulation rate of the *Salvia officinalis* extract in a gelatin/pectin system obtained by a complex coacervation process with a preliminary emulsification step was in the order of 96.57 ± 3.05%.

**Figure 2:**
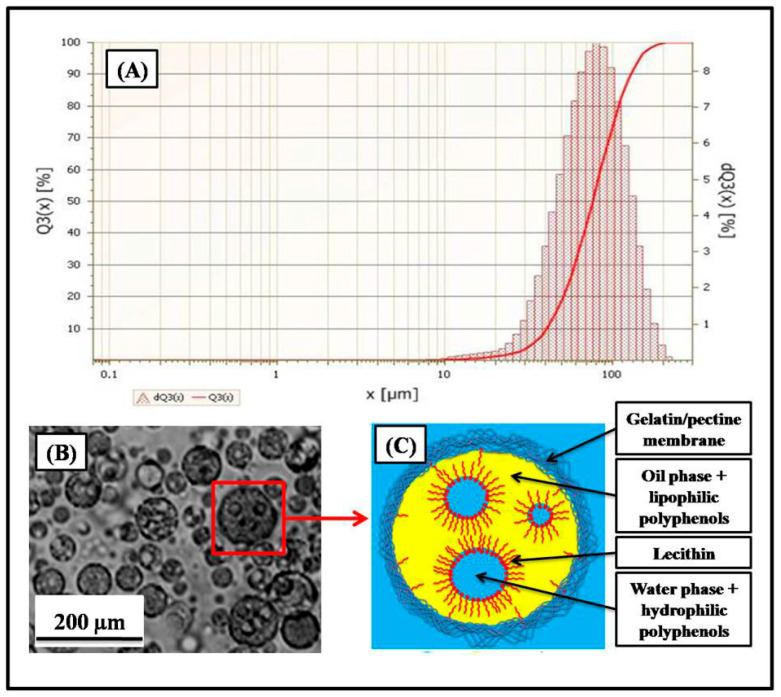
Structural characterization of microcapsules: (**A**) particle size distribution, (**B**) microphotograph of the microcapsules, and (**C**) molecular structure diagram of the microcapsules.

Figure 2A shows the particle size distribution of the microcapsules obtained by laser particle size measurement. The curve of the laser particle size analysis represents a single particle size distribution, and the average diameter of the microcapsules was in the order of 104.2 ± 7.5 µm.

Figure 2B shows a microphotograph of the microcapsules obtained (observation with the optical microscope, magnification ×40). The results showed individual microparticles of spherical shape with well-delimited walls contained inside small droplets.

Figure 2C shows a molecular structure diagram of the microcapsules obtained. The spherical microparticles were delimited by a polymeric membrane formed during the complex coacervation process between the two macromolecules, positively charged gelatin and negatively charged pectin. Inside the microparticles, there was an emulsion formed of two phases, water in oil (W/O), inverse emulsion, stabilized with lipophilic surfactant (lecithin). The aqueous phase (W) contains water-soluble bioactive molecules of encapsulated extract, and the organic phase (O) contains lipophilic bioactive molecules.

#### 3.2.2. In Vitro Dissolution Kinetics

**Figure 3:**
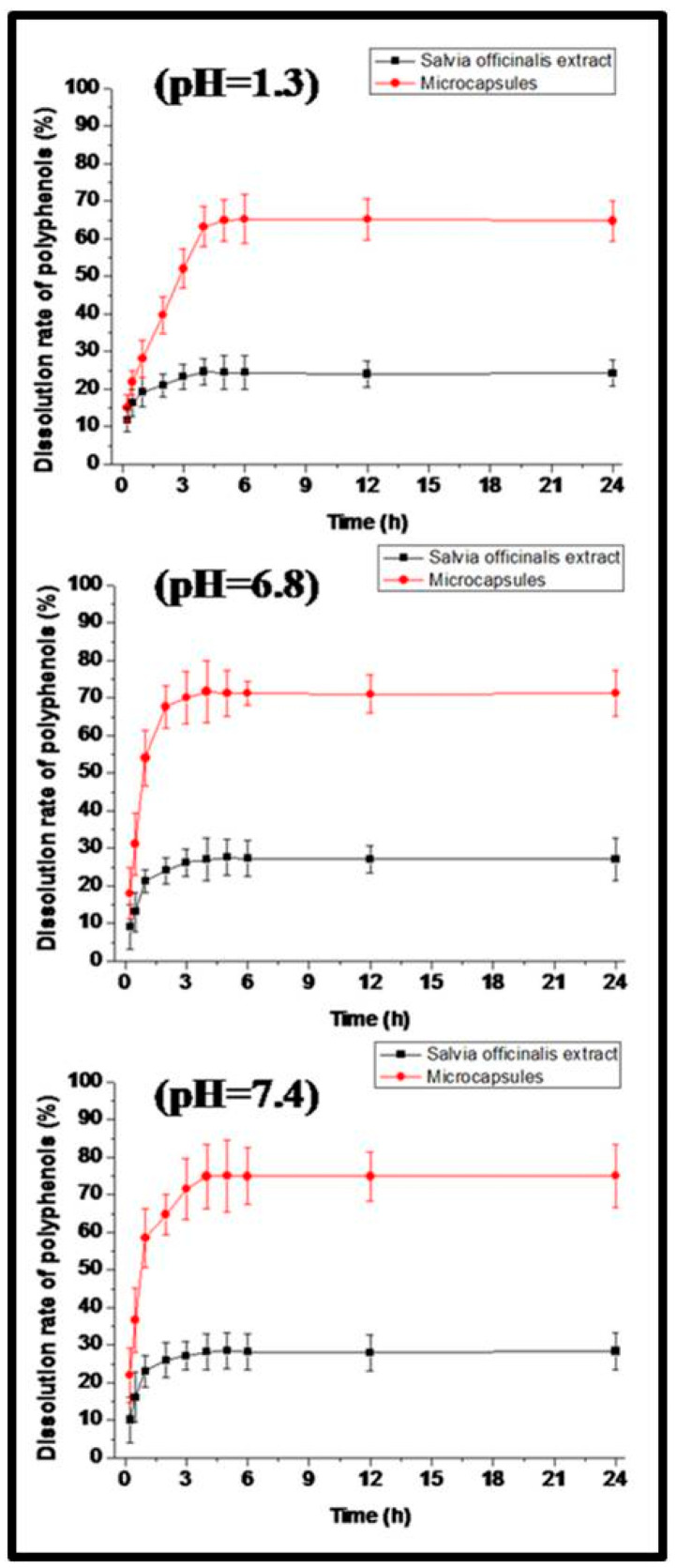
In vitro dissolution kinetics of microcapsules and non-encapsulated *Salvia officinalis* extract.

Figure 3 shows dissolution kinetics of the microcapsules and non-encapsulated Salvia officinalis extract. Maximum dissolutions (at pH = 1.3) of the free extract and microcapsules were detected as 24.62% and 64.82%, respectively. Maximum dissolutions (at pH = 6.8) of the free extract and microcapsules were 27.58% and 71.69%, respectively. Maximum dissolutions (at pH = 7.4) of the free extract and microcapsules were 28.56% and 74.99%, respectively.

#### 3.2.3. Evaluation of Ex Vivo Bioadhesion

**Figure 4:**
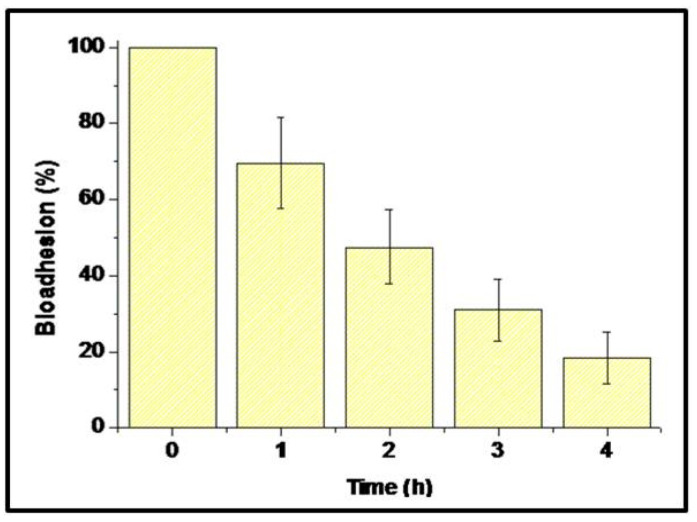
Ex vivo bioadhesion of microcapsules.

Figure 4 represents the results of ex vivo bioadhesion of the microcapsules. The results show bioadhesions of 69.54 ± 24.19 %, 47.51 ± 19.66 %, 31.07 ± 16.42 %, and 18.50 ± 13.23% after 1 h, 2 h, 3 h, and 4 h of evaluation, respectively.

### 3.3. Evaluation of Biological Activities

#### 3.3.1. In Vivo Evaluation of Anti-Ulcerogenic Activity

**Figure 5:**
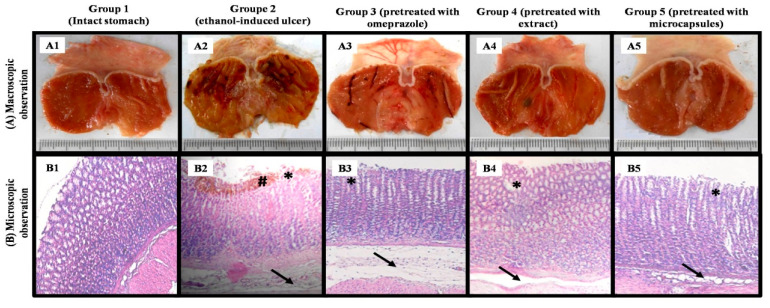
Macroscopic (**A**) and microscopic (**B**) structure of different stomachs: (**A1**,**B1**) physiologic group, (**A2**,**B2**) ethanol-induced group, (**A3**,**B3**) pretreated group with omeprazole, (**A4**,**B4**) pretreated group with extract, (**A5**,**B5**) pretreated group with microcapsules, (arrow) edema, (*) epithelial desquamations, and (#) bleeding.

Figure 5 shows the macroscopic (A) and microscopic (B) structure of different stomachs (physiological, ulcerated, and treated). Table 2 shows the macroscopic and microscopic characteristics of ulcers.

Ethanolic extract of *Salvia officinalis* showed a higher percentage of ulcer inhibition compared to the omeprazole reference drug. Actually, the percentages of inhibition by omeprazole and the ethanolic extract of *Salvia officinalis* were 53.85 ± 1.61% and 71.71 ± 2.43%, respectively.

The anti-ulcerogenic activity of this extract increased significantly after microencapsulation from 71.71 ± 2.43% to 89.76 ± 2.54%.

The macroscopic observation showed that the group 1 stomachs (physiological) were intact and did not show any lesions (Figure 5(A1)), whereas the group 2 stomachs (negative control) showed very serious extensive lesions on a large part of stomachs (the area of ulcer is very large) and significant bleeding (Figure 5(A2)). The other lots, lot 3 (treated with omeprazole) in Figure 5(A3), lot 4 (treated with extract) in Figure 5(A4), and lot 5 (treated with the microcapsules) in Figure 5(A5), showed fewer lesions, respectively, than lot 1 (negative control) and were closer to the physiological state (lot 1).

The microscopic observation of the stomachs of lot 3 (treated with omeprazole) and lot 4 (treated with the extract) showed significant edema of the sub-mucosa, weak epithelial desquamations, and glandular destruction, while no hemorrhages were detected (Figure 5(B3,B4)).

Lot 5 (treated with the microcapsules) showed a histological structure very close to normal with weak epithelial desquamations and slight edema of the sub-mucosa, with total absence of glandular destruction, infiltration of eosinophils, and hemorrhages (Figure 5(B5)).

#### 3.3.2. Evaluation of H^+^/K^+^ ATPase-Inhibiting Activity

**Figure 6:**
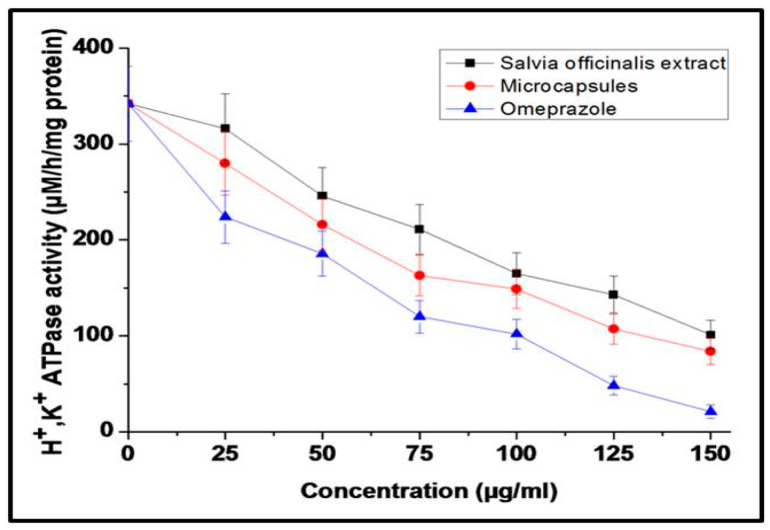
Evolution of H^+^/K^+^ ATPase-inhibiting activity as a function of concentration for the *Salvia officinalis* free extract, microcapsules, and omeprazole.

Figure 6 shows the evolution of H^+^/K^+^ ATPase-inhibiting activity as a function of concentration for the *Salvia officinalis* free extract, microcapsules, and omeprazole. The IC50 for the free *Salvia officinalis* extract, microcapsules, and omeprazole were 103.90 ± 10.44, 86.08 ± 8.69, and 57.43 ± 5.78 µM/h/mg of protein, respectively.

#### 3.3.3. Evaluation of Anti-*Helicobacter pylori* Activity

The MIC for *Helicobacter pylori* for the free *Salvia officinalis* extract, microcapsules, and metronidazole were all 50 µg/mL, whereas amoxicillin had a MIC of 0.01 µg/mL.

### 3.4. Discussion of Biological Activities and Biopharmaceutical Characteristics

The present study provides comprehensive evidence supporting the pharmacological potential of *Salvia officinalis* extract, particularly after its encapsulation in a gelatin/pectin coacervate system. The findings underscore the importance of optimized extraction, encapsulation, and delivery strategies to enhance bioactivity and site-specific action of bioactive phytomolecules.

The extraction yield of *Salvia officinalis* bioactive compounds (24.16 ± 2.05%) falls within the range reported for other phenolic-rich medicinal plants extracted under hydroalcoholic conditions. The HPLC/UV-DAD analysis revealed a well-defined polyphenolic profile dominated by flavonoids (rutin: 21.74%, quercitrin: 14.39%) and phenolic acids (chlorogenic acid: 7.43%, rosmarinic acid: 5.88%), together constituting approximately 49.44% of the extract. This composition is consistent with previous reports on *Salvia officinalis*, in which rutin and rosmarinic acid are commonly identified as key contributors to the plant’s antioxidant and anti-inflammatory effects. The significant presence of rutin and chlorogenic acid is noteworthy due to their known gastroprotective properties, which may explain the observed biological activities.

Encapsulation of the extract via complex coacervation resulted in an exceptionally high efficiency (96.57 ± 3.05%), indicating successful incorporation of the hydrophilic and lipophilic constituents into the biopolymer matrix. The average particle size (104.2 ± 7.5 µm) and spherical morphology with well-defined walls observed under optical microscopy support the suitability of the formulation for oral delivery. The structural model of the microcapsules (comprising a gelatin/pectin membrane surrounding a stabilized W/O emulsion) suggests efficient phase separation and entrapment of diverse bioactive constituents. This dual encapsulation system allows simultaneous protection and controlled release of both water- and lipid-soluble compounds, enhancing stability and bioavailability in the gastrointestinal environment.

Dissolution kinetics revealed a substantial improvement in release profiles for the encapsulated extract compared to the free extract across all the tested pH values. Notably, at pH 7.4, mimicking intestinal conditions, the microcapsules achieved 74.99% release versus 28.56% for the free extract. This enhanced release under intestinal pH can be attributed to the swelling and partial degradation of the biopolymeric matrix, facilitating drug diffusion. Furthermore, the ex vivo mucoadhesion assays demonstrated that the microcapsules retained up to 69.54 ± 24.19 % adhesion after 1 h, with gradual detachment over 4 h. Such prolonged residence time at the mucosal surface is advantageous for local delivery and sustained therapeutic action, especially in ulcerated or inflamed tissues.

The results of this study demonstrate a remarkable anti-ulcerogenic activity of the ethanolic extract of Salvia officinalis, significantly surpassing that of the reference drug omeprazole (71.71 ± 2.43% vs. 53.85 ± 1.61% ulcer inhibition). This superior efficacy is likely due to the synergistic effects of phenolic compounds, particularly rosmarinic acid and flavonoids, which are known to modulate oxidative stress and inflammatory pathways, as previously reported in recent studies using ethanol-induced ulcer models in rats [39]. Moreover, microencapsulation of the extract further enhanced its therapeutic effect, reaching an ulcer inhibition rate of 89.76 ± 2.54%. This finding underscores the role of microencapsulation in protecting bioactive compounds and enabling their controlled release, thereby improving their in vivo bioavailability and efficacy. These results are consistent with recent studies showing enhanced biological activities of various encapsulated plant extracts [40,41]. Histological analysis supported the pharmacological findings: the group treated with the microcapsules exhibited near-normal gastric mucosa, with minimal edema and desquamation, and no signs of hemorrhage or glandular damage. However, the study has some limitations. The model used involves acute gastric lesions induced by ethanol, which does not fully replicate the complex and multifactorial pathophysiology of chronic gastroduodenal ulcers, but the findings suggest potential translatability to human pathophysiology, given the conserved mechanisms involved in oxidative stress and inflammation.

The microcapsules also showed improved inhibition of H^+^/K^+^ ATPase activity (IC_50_ = 86.08 ± 8.69 µM/h/mg protein) compared to the free extract (IC_50_ = 103.90 ± 10.44 µM), approaching the efficacy of omeprazole (IC_50_ = 57.43 ± 5.78 µM). This enzyme inhibition is critical for acid suppression in ulcer therapy, suggesting that the microcapsules may act through a mechanism similar to PPIs. In parallel, both the extract forms exhibited moderate anti-H. pylori activity (MIC = 50 µg/mL), equivalent to metronidazole but significantly less potent than amoxicillin (MIC = 0.01 µg/mL). Nevertheless, the natural extract may serve as an adjunct to antibiotic therapy, particularly given the growing resistance to conventional drugs.

The formulation has improved anti-ulcerogenic properties of *Salvia officinalis* extract. Indeed, the formulation showed a greater in vivo gastroprotective effect and a greater inhibition of H^+^/K^+^ ATPase in vitro while preserving anti-*Helicobacter pylori* power.

The present study allowed us to propose three different mechanisms that can improve anti-ulcerogenic properties of *Salvia officinalis* extract formulation.

**Figure 7:**
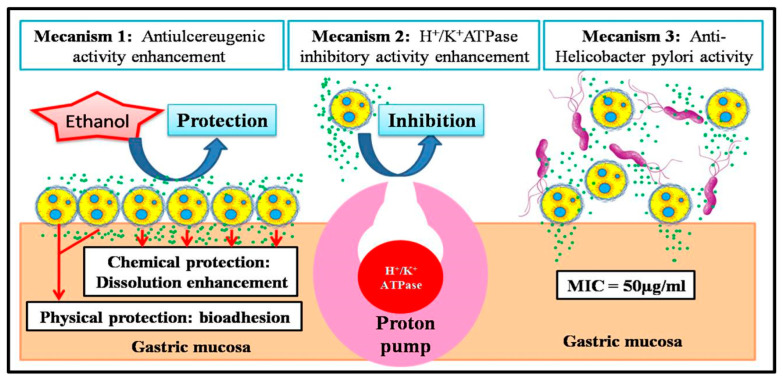
Representation of the mechanisms that permit the improvement of anti-ulcerogenic properties of *Salvia officinalis* extract formulation.

Mechanism 1 (increase in in vivo gastro-protective activity): The proposed mechanism for this improvement in the gastro-protective activity of sage extract after its microencapsulation was based on two phenomena (Figure 7).

The first phenomenon was bioadhesion; bioadhesive properties of the biopolymers composing the formulation (gelatin and pectin) [42,43] allow the microcapsules to adhere to the gastric mucosa and protect it physically against ulcerative agents (in our study, it was ethanol) [44].

The second phenomenon was the increase in solubility of the bioactive molecules contained in sage extract. Indeed, the increase in this in vitro dissolution after encapsulation of sage extract corresponds to the increase in in vivo bioavailability and thus to an increase in pharmacological properties [45].

Mechanism 2 (increase in in vitro H^+^/K^+^ ATPase-inhibiting activity): The increase in this activity after microencapsulation of sage extract was due to interaction increase between the microcapsules contained in the formulation and the proton pumps that carry H^+^/K^+^ ATPase enzymes (Figure 7). According to literature studies, small particles used in the delivery of drugs have a greater therapeutic effect than free drug molecules, because these particles have better penetration of living tissues and greater interaction with biological systems [46,47,48,49].

Mechanism 3 (preservation of anti-*Helicobacter pylori* activity): The sage extract and formulation have the same anti-*Helicobacter pylori* activity. This activity showed no variation as it was not dependent on physical factors such as solubility or bioadhesion, but rather on the nature of pharmacophores that act on *Helicobacter pylori*.

## 4. Conclusions

This study aimed to develop and evaluate a bioadhesive microparticulate system based on gelatin and pectin for the microencapsulation of *Salvia officinalis* ethanolic extract, which is rich in phenolic compounds (notably chlorogenic acid, rutin, rosmarinic acid, and quercitrin). The encapsulation was successfully achieved using an emulsion–coacervation technique, yielding high encapsulation efficiency (96.57 ± 3.05%) and uniform microparticles with a mean diameter of approximately 104.2 ± 7.5 µm. Microscopic and particle size analyses confirmed the spherical morphology and homogeneity of the microcapsules. The encapsulation enhanced the extract’s dissolution kinetics across simulated gastric and intestinal pH conditions (1.3, 6.8, and 7.4), and ex vivo tests confirmed excellent mucoadhesive properties, which are essential for prolonged residence time in the gastrointestinal tract. From a pharmacological perspective, microencapsulation also improved the in vivo anti-ulcerogenic activity of the extract, as well as its in vitro inhibitory effect on the H^+^/K^+^-ATPase enzyme, a key therapeutic target in acid-related disorders. Both the raw extract and its encapsulated form demonstrated significant anti-*Helicobacter pylori* activity. Taken together, these findings suggest that microencapsulation in a natural polymer matrix can be a promising strategy to optimize the gastroprotective potential of botanical extracts by improving their stability, bioavailability, and targeted delivery. However, further studies are needed to characterize the long-term stability of the formulation and to validate its efficacy and safety through in vivo pharmacokinetic and clinical investigations.

## Figures and Tables

**Table 1:** Experimental design of in vivo anti-ulcerogenic activity.

Groups	Nom	Number of Rats	Gavage at t_0_ (Treatment)	Gavage at t_0_ + 30 min (Ethanol)
Group 1	physiological	6	no	no
Group 2	negative control	6	no	yes
Group 3	positive control	6	omeprazole at 50 mg/kg	yes
Group 4	Treatment 1	6	*Salvia officinalis* extract (100 mg/kg)	yes
Group 5	Treatment 2	6	Microcapsules (equivalent to 100 mg/kg of *Salvia officinalis* extract)	yes

**Table 2:** Macroscopic and microscopic characteristics of ulcers.

Groups	Macroscopic		Microscopic				
	Percentages of Ulcer (%)	Percentages of Inhibitions (%)	Epithelial Desquamations	Glandular Destruction	Edema of the Sub-mucosa	Infiltration of Eosinophils	Hemorrhages
**Group 1**			-	-	-	-	-
**Group 2**	10.25 ± 0.17		+++	+++	+++	++	++
**Group 3**	4.73 ± 0.18 ^a^	53.85 ± 1.61	+	+	++	+	-
**Group 4**	2.9 ± 0.26 ^a,b^	71.71 ± 2.43	++	+	+++	+	-
**Group 5**	1.05 ± 0.25 ^a,b,c^	89.76 ± 2.54	+	-	+	-	-

^a^: highly significant results (*p* < 0.01) compared to group 2. ^b^: highly significant results (*p* < 0.01) compared to group 3. ^c^: highly significant results (*p* < 0.01) compared to group 4.

## Data Availability

The original contributions presented in this study are included in the article. Further inquiries can be directed to the corresponding author.
